# Mapping the patent landscape of synthetic biology for fine chemical production pathways

**DOI:** 10.1111/1751-7915.12401

**Published:** 2016-08-03

**Authors:** Pablo Carbonell, Abdullah Gök, Philip Shapira, Jean‐Loup Faulon

**Affiliations:** ^1^ Manchester Centre for Fine and Specialty Chemicals (SYNBIOCHEM)Manchester Institute of BiotechnologyUniversity of Manchester131 Princess StreetManchesterM1 7DNUK; ^2^ Manchester Institute of Innovation ResearchAlliance Manchester Business SchoolUniversity of ManchesterOxford RoadManchesterM13 9PLUK; ^3^ School of Public PolicyGeorgia Institute of Technology685 Cherry StreetAtlantaGA30332‐0345USA; ^4^ MICALIS InstituteINRADomaine de Vilvert78352Jouy en Josas CedexFrance

## Abstract

A goal of synthetic biology bio‐foundries is to innovate through an iterative design/build/test/learn pipeline. In assessing the value of new chemical production routes, the intellectual property (IP) novelty of the pathway is important. Exploratory studies can be carried using knowledge of the patent/IP landscape for synthetic biology and metabolic engineering. In this paper, we perform an assessment of pathways as potential targets for chemical production across the full catalogue of reachable chemicals in the extended metabolic space of chassis organisms, as computed by the retrosynthesis‐based algorithm RetroPath. Our database for reactions processed by sequences in heterologous pathways was screened against the PatSeq database, a comprehensive collection of more than 150M sequences present in patent grants and applications. We also examine related patent families using Derwent Innovations. This large‐scale computational study provides useful insights into the IP landscape of synthetic biology for fine and specialty chemicals production.

## Introduction

Efficient and sustainable microbial production through cell factory synthetic biology is a promising enabling technology in the transition from an economy based on fossil fuels towards an economy based on renewable biomass resources (SBLC, 2016). The synthetic biology of fine and speciality chemicals is a biomanufacturing technology that, by 2020, is envisioned as providing cheaper economical, predictable and sustainable routes to an enlarged portfolio of novel, diverse, and previously expensive products in many sectors including cosmetics, flavors, polymers, and pharmaceuticals (Carbonell *et al*., 2016; Breitling and Takano 2016). A roadmap to commercialization of such products will need to both integrate and navigate the scientific and technological knowledge accumulated in patents into the biodesign process, so as to reduce transaction costs and lower the risk of failure as well as to maximize the innovation capabilities of the synthetic biology pipeline. One reported estimate suggests that by 2025 bio‐production will represent up to 28% of the global chemical market ($614Bm) (USDA 2008). Industrial bio‐production is nevertheless a complex process whose analysis requires multi‐scale approaches integrating cell, bioprocess, and ecosystem models with economic, innovation and policy considerations. Recent advances in metabolic engineering and synthetic biology have led to an increase in the portfolio of both key building block chemicals and fine chemicals that can be microbially produced from biomass feedstock (Curran and Alper, 2012). Emerging computation platforms for synthetic biology (Carbonell *et al*., 2016b) in combination with rapid DNA engineering and assembly techniques (Casini *et al*., 2015) are contributing to streamline metabolic pathway design and strain engineering. Synthetic biology techniques that insert standardized and characterized genetic parts are used to regulate and fine‐tuning the engineered biosynthetic pathways in the chassis (Chao *et al*., 2015). Nowadays, the establishment of synthetic biology bio‐foundries enables the application of automated design–build–test–learn cycles to microbial cell factories so that natural pathways can be refactored and novel reactions and products can be discovered (Hara *et al*., 2014; Carbonell *et al*., 2016a).

Yet, despite the increasing number of small molecules that are bio‐produced, the process still remains a complex system, costly and rather slow. For instance, the metabolic engineering of artemisinic acid is claimed to have taken over 200 person‐years to date (Nielsen and Keasling, 2016). The challenge of sustainable chemical production, thus, needs to be addressed not only by considering the different temporal and spatial scales that are present in microbial production bioprocesses (Zhuang *et al*., 2013), but also by monitoring innovation (Kahl and Endy, 2013) to define multi‐objective goals compatible with a more rational use of biomass. Here, we perform a patent analysis of biosynthetic pathways for fine chemicals to explore the innovation landscape of metabolic design for microbial synthetic production. Our study compared the reachable metabolic space in the chassis *Escherichia coli*, based on our current knowledge of enzyme sequences present in biochemical routes, with the set of sequences disclosed in patents since 1970 (Jefferson *et al*., 2015).

Although multiple factors are involved in the successful commercialization of scientific and technological knowledge, the accumulation and analysis of intellectual property (IP) through patenting offers important signals as to targets that might subsequently have exploitable value in biotechnology and synthetic biology (Petruzzelli *et al*., 2015; see also Bubela *et al*., 2013). Performing patent analyses in emerging technologies such as synthetic biology is challenging, as specific classifications are lacking or at best lagging in the international patent classification system. Prior studies (e.g. van Doren *et al*., 2013) have attempted to define synthetic biology patents using selected keywords. However, such approaches face problems of both precision and recall. In this study, we put forward a novel approach by analysing chemical pathways. We focus on a specific type of synthetic biology application, that is, expression of chemical pathways for the production of fine and speciality chemicals in cell factories. Moreover, we restricted our study to those cases corresponding to synthetic pathways that are not naturally produced in the chassis. Non‐native biosynthetic pathways are of special interest since the expression in the heterologous host can be performed through a full refactoring of the pathway and combinatorial biosynthesis (Luo *et al*., 2015). This focused approach allows us to develop a more finely tuned search methodology: we define our strategy in terms of disclosed sequences encoding biosynthetic enzymes that belong to pathways not present in the chassis rather than focusing on their chemical targets (Papadatos *et al*., 2016). This approach can suffer in some cases from low precision in instances, where these sequences are mentioned in the patent for some other reason not related to bioproduction. Nevertheless, this issue does not invalidate the basic approach of this study of searching and analysing heterologous pathways in patents through their associated enzymes. Notably, this synthetic biology application has been already present in some form since earlier biotechnology applications such as genome and metabolic engineering. Hence, we can assess the impact that the advent of synthetic biology and engineering biology technologies has exerted in metabolic design and the development of cell factories, and also in the associated IP landscape.

## Results and discussion

We screened for disclosed sequences in patent documents since 1970 (Jefferson *et al*., 2015) that matched sequences encoding enzymes in biosynthetic pathways for fine and speciality chemicals in metabolic databases (Alcántara *et al*., 2012; Caspi *et al*., 2012; Moretti *et al*., 2016). (See Data S1 for discussion and sources of patent documents and patent families.) The aim was to identify cases potentially useful for synthetic biology applications involving the expression of gene sequences imported from foreign organisms into a chassis host. Our reference chassis is *E. coli* and therefore the study focused on enzymes that were not natively found in that chassis.

**Figure 1:**
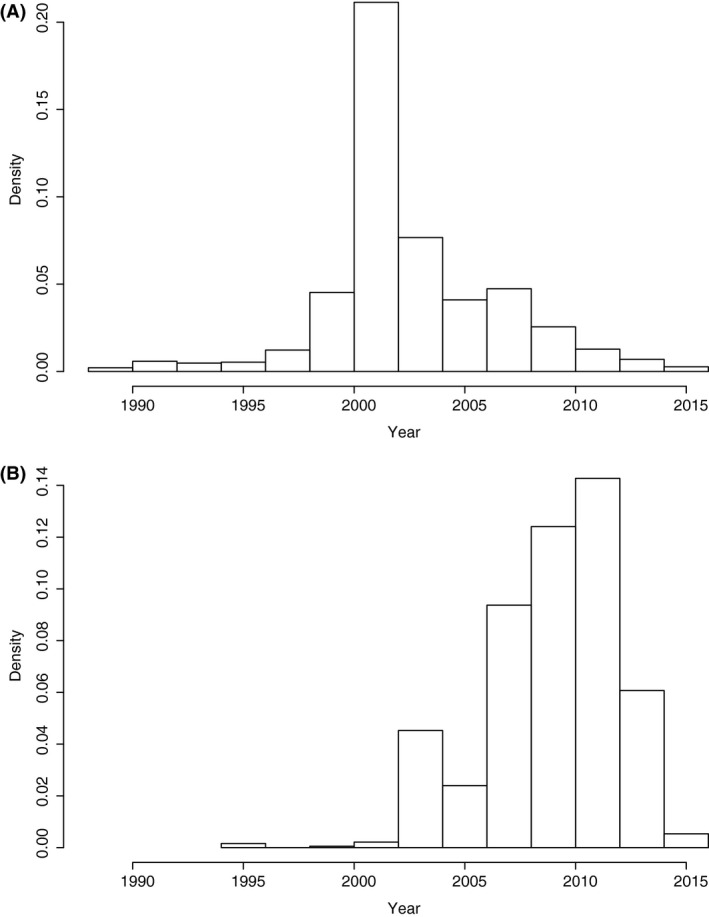
Incidence of pathways in patent documents per year: (A) frequency of year of first occurrence of a pathway in analysed patents; (B) median of occurrence of the pathway.

We traced references to enzyme sequences in patents to as early as 1988, where we found a reference in patent WO 1988007079 A1 to the sequence of a tyrosinase (EC 1.14.18.1) from *Streptomyces* catalyzing the oxidation of L‐tyrosine into L‐DOPA (Satoh *et al*., 2012). Figure 1A shows the years of first occurrence of pathways in patent documents, with a maximum incidence centred around the year 2002. A different result emerged when we consider the median year of occurrence of each pathway, as shown in Fig. 1B. In this case, we can see a consistent growth in time in the number of times that a pathway appears in a patent document, at least up to 2015. Note that there is a lag between patent application, publication, and inclusion in databases, so records for most recent years are not yet complete.

**Figure 2:**
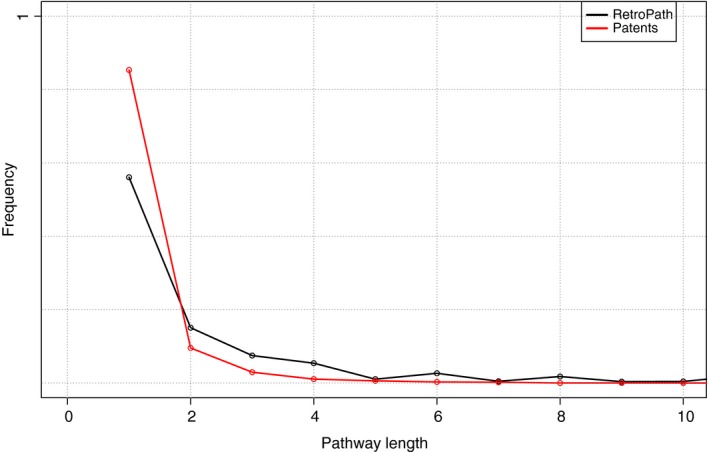
Frequency of pathway lengths found in patent documents compared with reachable pathways in *Escherichia coli* according to RetroPath.

As anticipated, biosynthetic pathways involving low number of enzymes have higher incidence in patent documents than in the full catalogue of pathways amenable to metabolic engineering, as shown in Fig. 2. We can expect this result, as the expression and balancing of heterologous pathways in chassis introduces a metabolic burden, among other issues, that often increases with the number of foreign enzymes that are expressed. When alternative pathways exist for the production of the same compound, the first choice for a synthetic pathway may be the shortest one. Therefore, the rate of success for the expression of a heterologous pathway generally decreases with pathway length. In most cases, a single heterologous pathway was identified in the patent document, although in some cases, patent documents contained enzymes associated with multiple pathways (Fig. S1). Similarly, the number of target compounds that could be produced through the pathways found in patent documents was often found to be higher than one (Fig. S2). However, these numbers are influenced by the fact that the presence of one pathway implies also those pathways producing each heterologous intermediate across the biosynthetic route. The previous analysis, thus, reflects different perspectives as both pathways themselves imply the production of intermediates and also multiple alternative pathways may be available for producing a target compound.

Enumerating all pathways that can produce a target compound is not trivial, as it requires specialized algorithms. Several techniques exist based on graph theory, elementary flux modes, minimal cut sets, as well as others (Carbonell *et al*., 2012). We have considered here pathways enumerated by the RetroPath algorithm based on elementary flux modes (Carbonell *et al*., 2012, 2014). Once the total number of alternative pathways has been determined, several biotechnological considerations can lead to alternative choices depending on the expected pathway performance. Several criteria exist for ranking and pathway selection in addition to pathway length considerations, including thermodynamics feasibility, theoretical yield and enzyme performance (Carbonell *et al*., 2016a,b). Furthermore, we consider here that the choice of a pathway can also be influenced by its intellectual property rights protection. Interestingly, we found cases where the number of alternative pathways per target compounds found in patent documents went up to 13 alternative pathways, which implies a high degree of exploration and innovation in the search for the optimal pathway to produce a desired compound (Fig. S3).

Our screening for biosynthetic pathways in patent documents allowed us to gain insights into the landscape of chemicals of interest. We looked at the percentage of chemical classes whose biosynthetic pathways were identified in the sequence in pathways. We focused on some of the classes of higher interest. Table 1 provides a list for the selected classes based on the ChEBI ontology (Hastings *et al*., 2013) sorted by the number of compounds in our dataset of reachable heterologous compounds. We also looked at the organism whose sequence appeared more often for each class of compounds. We found, for instance that 36% of terpenoids appeared in patents and that *Arabidopsis thaliana* is its main source organism. Similarly, 15% and 28% of alkaloids and flavonoids considered as pathway‐reachable were found in patent documents respectively. Their main sources were *Coffea arabica* for alkaloids and *Medicago sativa* for flavonoids. Looking at the average market price of available compounds in the selected classes, we found that terpenoids found in patent documents had the greatest market value, which was considerable higher than the overall average market value of these compounds. (Aldrich Market Select was used to query market prices by compound chemical structure.) Flavonoids in patent documents also had a higher market value than their average market value, which was the opposite in the case of alkaloids. These results are helpful in informing consideration of the pricing implications of patented synthetic biology compounds compared with chemical and natural production. It should be stressed that this study was focused on compounds for which biosynthetic pathways are known and their enzyme sequences reported in databases. This means that the market values in Table 1 should be updated as long as novel sequences leading to additional, potentially more valuable pathways and compounds are discovered.

**Table 1:** Number of compounds and their average price found in patent documents in comparison with the reachable metabolic space and average market price (USD/10 mg) for available compounds

Class	Frequency	In path	All	Top organism	Avg. price all	Avg. price in path
Lipids	52.40	523	998	*Arabidopsis thaliana*	429.38	771.64
Carbohydrates	52.50	304	579	*A. thaliana*	494.19	568.08
Terpenoids	36.06	163	452	*A. thaliana*	643.06	1709.32
Phenols	46.51	180	387	*A. thaliana*	109.82	58.30
Hydrocarbons	81.19	246	303	*Abies grandis*	178.49	255.49
Flavonoids	27.54	76	276	*Medicago sativa*	273.40	404.81
Alcohols	55.07	152	276	*A. thaliana*	701.40	1159.63
Ketones	36.60	97	265	*M. sativa*	244.71	464.80
Amides	55.14	134	243	*A. thaliana*	70.52	60.98
Benzenes	33.01	69	209	*A. thaliana*	33.69	52.98
Carboxamides	55.90	109	195	*A. thaliana*	77.40	60.98
Benzopyrans	25.40	48	189	*M. sativa*	347.90	388.97
Phenylpropanoids	30.65	57	186	*A. thaliana*	216.75	269.63
Cyclic ketones	29.17	42	144	*M. sativa*	267.65	351.17
Diterpenoids	24.82	35	141	*Abies balsamea*	25.24	25.00
Alkaloids	15.91	21	132	*Coffea arabica*	41.94	14.14
Monoterpenoids	14.96	19	127	*Ocimum basilicum*	24.54	25.00
Polyols	53.33	64	120	*Homo sapiens*	107.02	78.40
Benzenediols	55.46	66	119	*Pseudomonas putida*	70.87	144.97
Primary alcohols	62.04	67	108	*A. thaliana*	1117.14	1680.94
Purines	51.61	48	93	*A. thaliana*	28.11	19.57
Benzopyrroles	72.37	55	76	*A. thaliana*	34.79	37.00
Sesquiterpenoids	58.11	43	74	*Streptomyces avermitilis*	2501.44	3717.33
Triterpenoids	61.67	37	60	*A. thaliana*	129.35	126.20
Monoterpenes	94.83	55	58	*A. grandis*	19.67	19.67
Hydroxyflavanones	58.49	31	53	*M. sativa*	263.96	351.17

**Figure 3:**
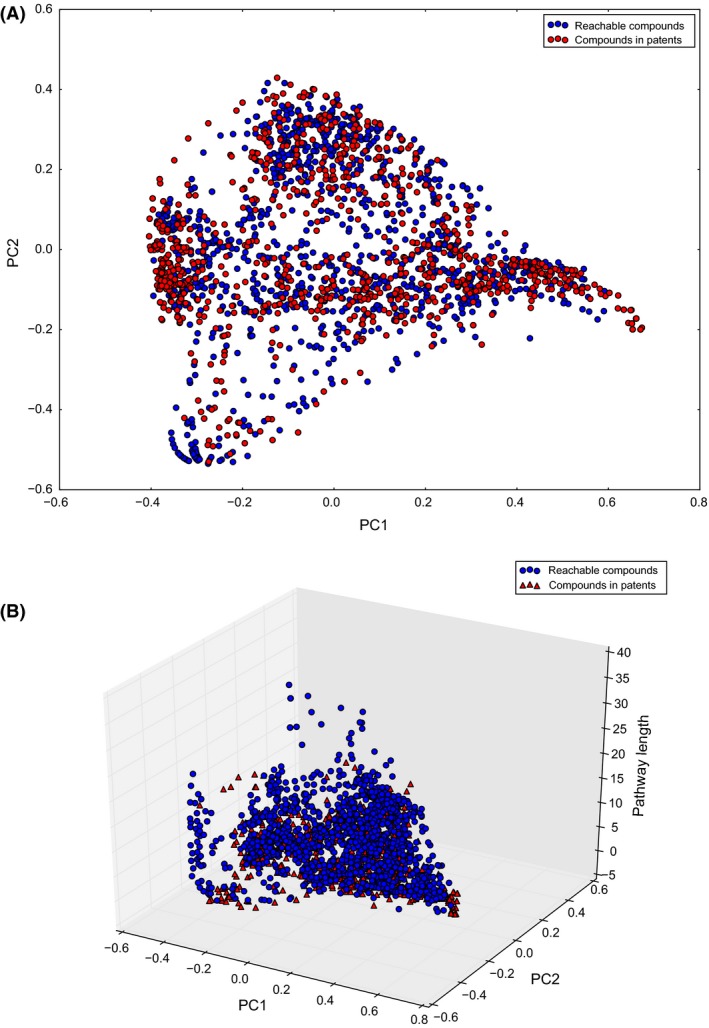
Distribution of compounds in patents in the reachable chemical space: (A) chemical space of reachable compounds (blue) highlighting those compounds whose pathways were found in patent documents (red); (B) chemical space of reachable compounds (blue) and compounds in patents (red) plotted with a third axis representing pathway length.

We also probed the chemical space covered by pathways found in patents in comparison with the area covered by the full reachable chemical space. The main question was to explore if there were privileged regions in the reachable space that were enriched in compounds present in patents, while others had less coverage and therefore could potentially constitute target compound classes in terms of innovation. Figure 3A shows the covered areas of the chemical space by the set of reachable compounds and the ones in patent documents. This representation is based on chemical descriptors, as described in supporting information, and it is generally used to analyse a set of compounds in terms of their chemical similarity. Interestingly, we observed that patent compounds tend to appear clustered at some specific regions, whereas other areas of the reachable space still remain basically under‐explored. One of the factors that may be associated with areas of lesser coverage is pathway length, as we previously discussed. Pathway length is arguably one of the most important limiting factors for heterologous pathway expression. As shown in Fig. 3B, when we added a third dimension that considered pathway length to the plot, we see that a considerable number of pathways that did not appear in patents (blue dots) belong to pathways involving multiple steps.

**Figure 4:**
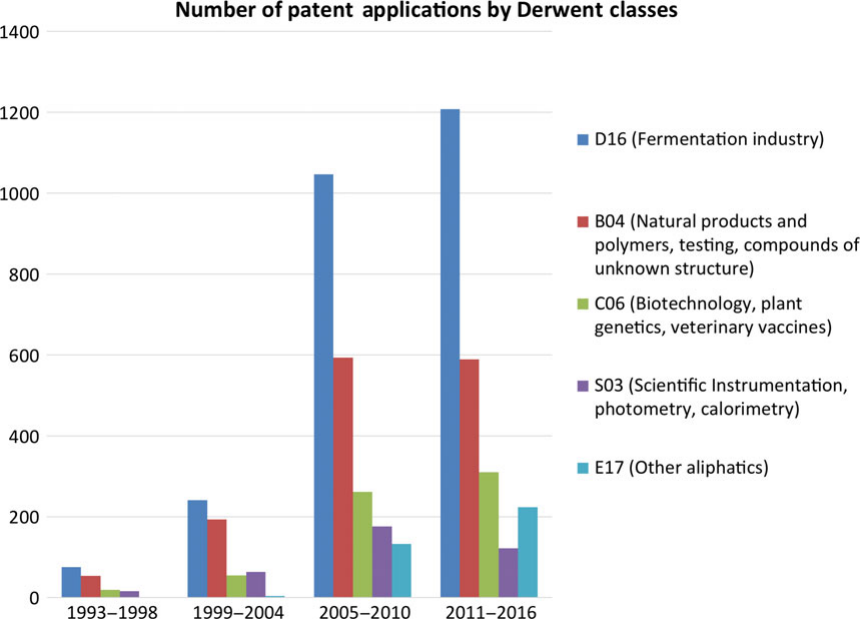
Number of Patents by Derwent Classes. Source: own calculation based on the Derwent Innovation Index (DII). A patent can be classified in more than one Derwent Class, thus total exceed 100%.

We further analysed the organizations and the targets involved in the patenting of synthetic biology for fine chemical production by linking our chemical pathways database to the Derwent Innovation Index Database. This analysis focused on patent families each of which can contain multiple patent documents (see Data S1 for more details). We find modest levels of patenting of fine chemical production pathways in the 1990s, with some tail‐off in the early 2000s. From the late 2000s, however, there was a rapid growth in the number of patent applications identified in Derwent, peaking at around 500 patents per year in recent periods (Fig. S4). Based on Derwent patent classes, the vast majority of patents (more than 90%) are classed in fermentation sectors, while natural products and polymers in pharmaceuticals (around 52%), biotechnology, plant genetics, veterinary vaccines (22%) and scientific instrumentation (around 14%) are the other leading classes (Table 2). While fermentation classifications have been maintained at high levels the past three decades, biotechnology in agricultural chemicals increased modestly and natural products and polymers decreased significantly (Fig. 4).

**Table 2:** Leading Derwent classifications of synthetic biology fine chemical patents

Derwent classifications (Classes)	Number of patents	Percentage of all patents
D16 (Fermentation industry)	1990	94%
B04 (Natural products and polymers, testing, compounds of unknown structure)	1100	52%
C06 (Biotechnology, plant genetics, veterinary vaccines)	469	22%
S03 (Scientific Instrumentation, photometry, calorimetry)	294	14%
E17 (Other aliphatics)	290	14%
P13 (Plant culture, dairy products)	243	11%
D13 (Other foodstuffs and treatment)	230	11%
B05 (Other organics – aromatics, aliphatic, organo‐metallics)	179	8%
T01 (Digital Computers)	130	6%
A41 (Monomers, Condensants; see also Section E)	103	5%

The leading patent assignees in our dataset comprise large commercial conglomerates active in a range of industries. No single organizations held more than 5% of our patent dataset. There is an evident specialization in terms of application areas by Derwent classes. While almost all leading firms have patents in fermentation sectors, companies with strong chemical industry capabilities such as Du Pont, BASF, Bayer as well as firms operating mainly in agri‐food such as Ajinomoto and Monsanto patented in the general category of “biotechnology, plant genetics, and veterinary vaccines.” On the other hand, a number of leading firms specialized in other applications areas; for instance, Butamax focused on fuels, Goodyear patented on monomers and condensants while natural products and polymers were targeted by Mitsui and Mitsubishi (Table 3). Universities, government and other research labs are also active in patenting through synthetic biology pathways. As with commercial organizations, most applications fall into fermentation, natural products and polymer classifications (Table 4).

**Table 3:** Leading commercial patenting organizations and Derwent classifications of their synthetic biology fine chemical patents

Organization	Number of patents	Share of patents in the dataset	Leading Patent Classes and their share in organization's patents in the dataset
DU PONT	88	4.0%	99%: D16 (Fermentation industry)
			41%: E17 (Other aliphatics)
			32%: C06 (Biotechnology, plant genetics, veterinary vaccines)
BASF	77	3.5%	100%: D16 (Fermentation industry)
			70%: C06 (Biotechnology, plant genetics, veterinary vaccines)
			53%: P13 (Plant culture, dairy products)
AJINOMOTO	58	2.7%	100%: D16 (Fermentation industry)
			62%: B05 (Other organics ‐ aromatics, aliphatic, organo‐metallics.)
			36%: D13 (Other foodstuffs and treatment)
DSM IP ASSETS	57	2.6%	96%: D16 (Fermentation industry)
			36%: D13 (Other foodstuffs and treatment)
			33%: B04 (Natural products and polymers, testing, compounds of unknown structure)
BUTAMAX ADVANCED BIOFUELS	50	2.3%	100%: D16 (Fermentation industry)
			75%: E17 (Other aliphatics)
			33%: H06 (Gaseous and liquid fuels including pollution control)
BAYER	46	2.1%	91%: D16 (Fermentation industry)
			72%: C06 (Biotechnology, plant genetics, veterinary vaccines)
			35%: P13 (Plant culture, dairy products)
MITSUI & TORAY	36	1.6%	81%: D16 (Fermentation industry)
			38%: E16 (Aliphatics containing N and/or halogem)
			31%: B04 (Natural products and polymers, testing, compounds of unknown structure)
MONSANTO	33	1.5%	97%: D16 (Fermentation industry)
			94%: C06 (Biotechnology, plant genetics, veterinary vaccines)
			64%: P13 (Plant culture, dairy products)
GOODYEAR TIRE & RUBBER CO	24	1.1%	100%: D16 (Fermentation industry)
			58%: E17 (Other aliphatics)
			54%: A41 (Monomers, Condensants)
MITSUBISHI	22	1.0%	41%: D16 (Fermentation industry)
			18%: T01 (Digital Computers)
			14%: B04 (Natural products and polymers, testing, compounds of unknown structure)

**Table 4:** Leading non‐commercial patenting organizations and Derwent classifications of their synthetic biology fine chemical patents

Organization	Number of patents	Share of patents in the dataset	Leading Patent Classes and their share in organizations identified patents
UNIV CALIFORNIA	32	1.5%	97%: D16 (Fermentation industry)
			56%: B04 (Natural products and polymers, testing, compounds of unknown structure)
			47%: E17 (Other aliphatics)
CNRS	17	0.8%	100%: D16 (Fermentation industry)
			76%: B04 (Natural products and polymers, testing, compounds of unknown structure)
			23%: C06 (Biotechnology, plant genetics, veterinary vaccines)
TECHNION	12	0.5%	100%: D16 (Fermentation industry)
			92%: B04 (Natural products and polymers, testing, compounds of unknown structure)
			25%: S03 (Scientific Instrumentation, photometry, calorimetry)
INSERM	11	0.5%	100%: D16 (Fermentation industry)
			91%: B04 (Natural products and polymers, testing, compounds of unknown structure)
			27%: S03 (Scientific Instrumentation, photometry, calorimetry)
UNIV OSAKA	11	0.5%	100%: D16 (Fermentation industry)
			91%: B04 (Natural products and polymers, testing, compounds of unknown structure)
			18%: S03 (Scientific Instrumentation, photometry, calorimetry)
HEBREW UNIV JERUSALEM	10	0.5%	100%: D16 (Fermentation industry)
			60%: B04 (Natural products and polymers, testing, compounds of unknown structure)
			60%: C06 (Biotechnology, plant genetics, veterinary vaccines)
UNIV ILLINOIS	10	0.5%	100%: D16 (Fermentation industry)
			70%: B04 (Natural products and polymers, testing, compounds of unknown structure)
			40%: E17 (Other aliphatics)
UNIV KYOTO	10	0.5%	90%: D16 (Fermentation industry)
			50%: B04 (Natural products and polymers, testing, compounds of unknown structure)
			30%: S03 (Scientific Instrumentation, photometry, calorimetry)
UNIV KYUSHU	10	0.5%	100%: D16 (Fermentation industry)
			60%: B04 (Natural products and polymers, testing, compounds of unknown structure)
			40%: S03 (Scientific Instrumentation, photometry, calorimetry)
HARVARD UNIV	9	0.4%	89%: D16 (Fermentation industry)
			89%: B04 (Natural products and polymers, testing, compounds of unknown structure)
			44%: C06 (Biotechnology, plant genetics, veterinary vaccines)

These results present a novel way to develop new perspectives on the patenting landscape of synthetic biology, in this case focused on biosynthetic pathways for fine and speciality chemicals. The approach provides valuable information that can help in the design process, as it can serve to select targets of interest as well as to define criteria for enzyme selection in designated pathways in chassis organisms. Ultimately, these data may serve to develop machine‐learning approaches to predict best target compounds and enzymes according to innovation criteria. There are synthetic biology applications that have not been fully covered in this patent study, as long as they are not directly related to biosynthetic pathways. Our approach can be extended to the screening of other synthetic biology‐relevant sequences such as those deposited at public registries of biological parts, extending in that way the study to other genetic parts of synthetic biology devices and systems, such as regulatory and sensing components.

## Conflict of interest

None declared.

## Supporting information


**Fig. S1.** Number of different pathways per patent document.
**Fig. S2.** Number of different target compounds per patent document.
**Fig. S3.** Number of alternative pathways per compound.
**Fig. S4.** Number of patents by application year.
**Data S1.** Experimental procedures.
