# Supplementary material for: Mapping the patent landscape of synthetic biology for fine chemical production pathways
Source: Microb Biotechnol. 2016 Aug 3;9(5):687–95. doi: 10.1111/1751-7915.12401 (PMC4993189; doi:10.1111/1751-7915.12401)
Supplement: Supplementary file 1 — Fig. S1. Number of different pathways per patent document. Fig. S2. Number of different target compounds per patent document. Fig. S3. Number of alternative pathways per compound. Fig. S4. Number of patents by application year. Data S1. Experimental procedures. [file MBT2-9-687-s001.pdf]

Pablo Carbonell, Abdullah Gök, Philip Shapira, and Jean-Loup Faulon.

Mapping the patent landscape of synthetic biology for fine chemical production pathways.

## Supporting information

### Description

**Experimental procedures**

**Fig. S1.** Number of different pathways per patent document.

**Fig. S2.** Number of different target compounds per patent document.

**Fig. S3.** Number of alternative pathways per compound.

**Fig. S4.** Number of patents by application year.

## Experimental procedures

Annotated sequences were obtained from PatSeq database (Jefferson *et al.*, 2015). This database is a comprehensive collection of patent data spanning the period from 1970 to 2016. At the time of the writing, this database contains 337,362 amino acids sequences in applications, 121,488 in grants, 2013,149,589 nucleotide sequences in patent applications and 50,971,017 from worldwide applications, organized in jurisdictions. We used Metanetx (Moretti *et al.*, 2016) as reference database for metabolic reactions and chemicals. Metanetx is a meta-database that reconciliates into a single reference name space both reactions and metabolites extracted from main metabolic databases such as KEGG, Metacyc, Rhea or Reactome. We used Metanetx cross-link annotations for reactions in order to obtain enzyme Uniprot identifiers annotated for the reactions in Rhea (Alcantara *et al.*, 2012) and Metacyc (Caspi *et al.*, 2012). In total 208,980 sequences from 5388 organisms were associated to 7793 reactions in the database. The sequences in our metabolic

database were then searched in the entire collection of sequences in PatSeq database by using the sequence alignment tool BLAST.

Metabolic pathways producing heterologous compounds in the chassis were determined using our RetroPath algorithm (Carbonell *et al.*, 2014). The algorithm models enzymes in an extended metabolic space that consider not only native reactions but also reactions that can promiscuously process the enzyme (a detailed explanation of the method can be found in (Carbonell *et al.*, 2014) and references therein). Starting from a chassis organism like E. coli, RetroPath will enumerate all reachable compounds and associated pathways by importing heterologous enzymes.

In order to determine if a pathway was present in a patent, we considered the set of enzymes catalyzing the reactions in the pathway. If a given patent mentioned simultaneously all sequences encoding enzymes in the pathway with similarity greater than 0.8 according to BLAST, we assumed that the pathway for the compound appears in the patent. In total, we found 91972 hits.

Compound classes were based on ChEBI ontologies (Hastings *et al*., 2013). ChEBI ontology provides a structured classification of chemical entities. Its structure differs from a simple taxonomy, but a directed acyclic graph allowing a chemical entity to have many parent terms. Compound price quotes were obtained from Aldrich Market Select (http://aldrichmarketselect.com) (retrieved in May 12th, 2016). When multiples quotes were available from different vendors, we selected the lowest one.

In order to analyze the chemical space, we mapped each compound into the descriptor space given by its stereo molecular signatures (Carbonell *et al*., 2013), which are a specialized version of extended connectivity fingerprints that decompose a molecular structure into its elementary connectivity subgraphs. These descriptors allow performing similarity analysis between chemicals. We computed the set of descriptors up to atomic vicinity of 8 and we applied principal component analysis.

We searched patent documents obtained from the PatSeq database in the Derwent Innovations Index (DII) to study the evolution, scope, focus and actor space of the innovative activity in synthetic biology. DII is a reference database for studying inventive and innovative activity through patents as i) it is based on family of patents rather than patent documents and also ii) it provides a wide range of information for each patent family. Based on the search of patent documents, our DII patent family database comprised 2,118 records.

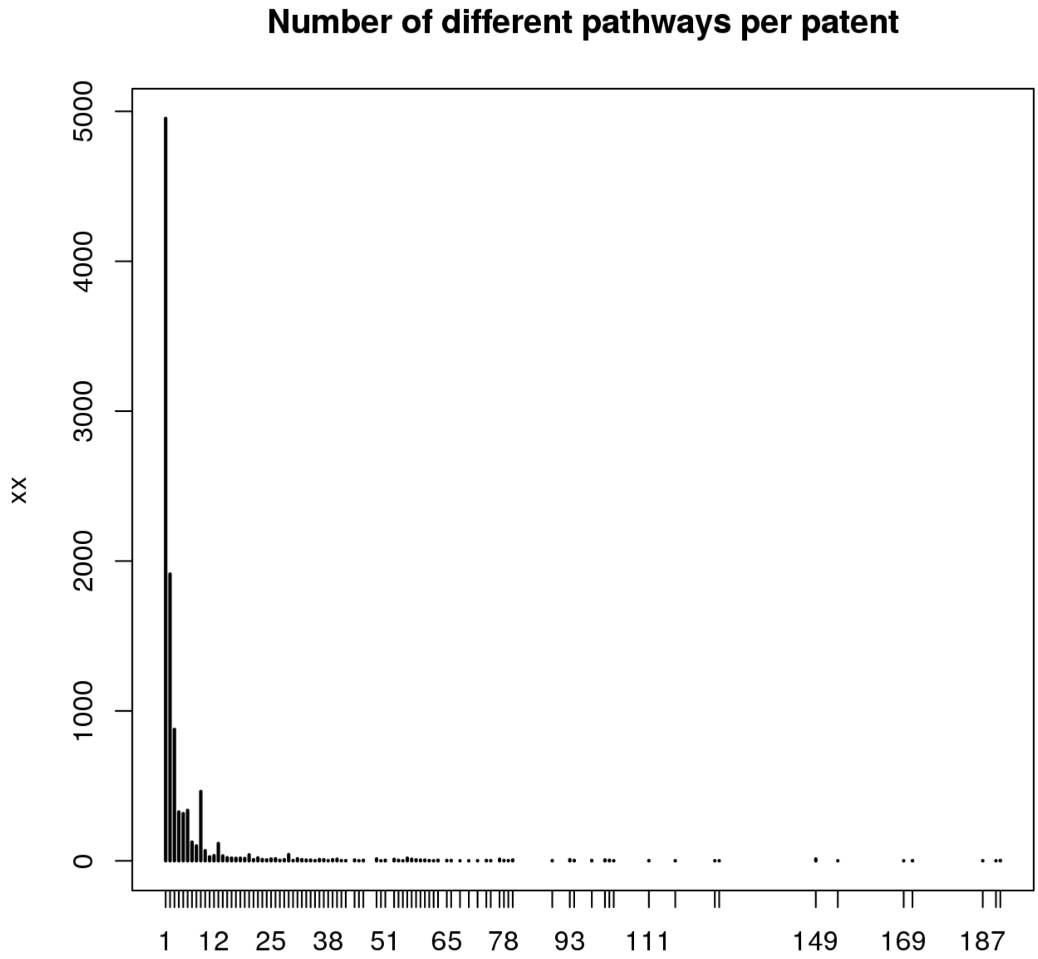


**Fig. S1.** Number of different pathways per patent document.

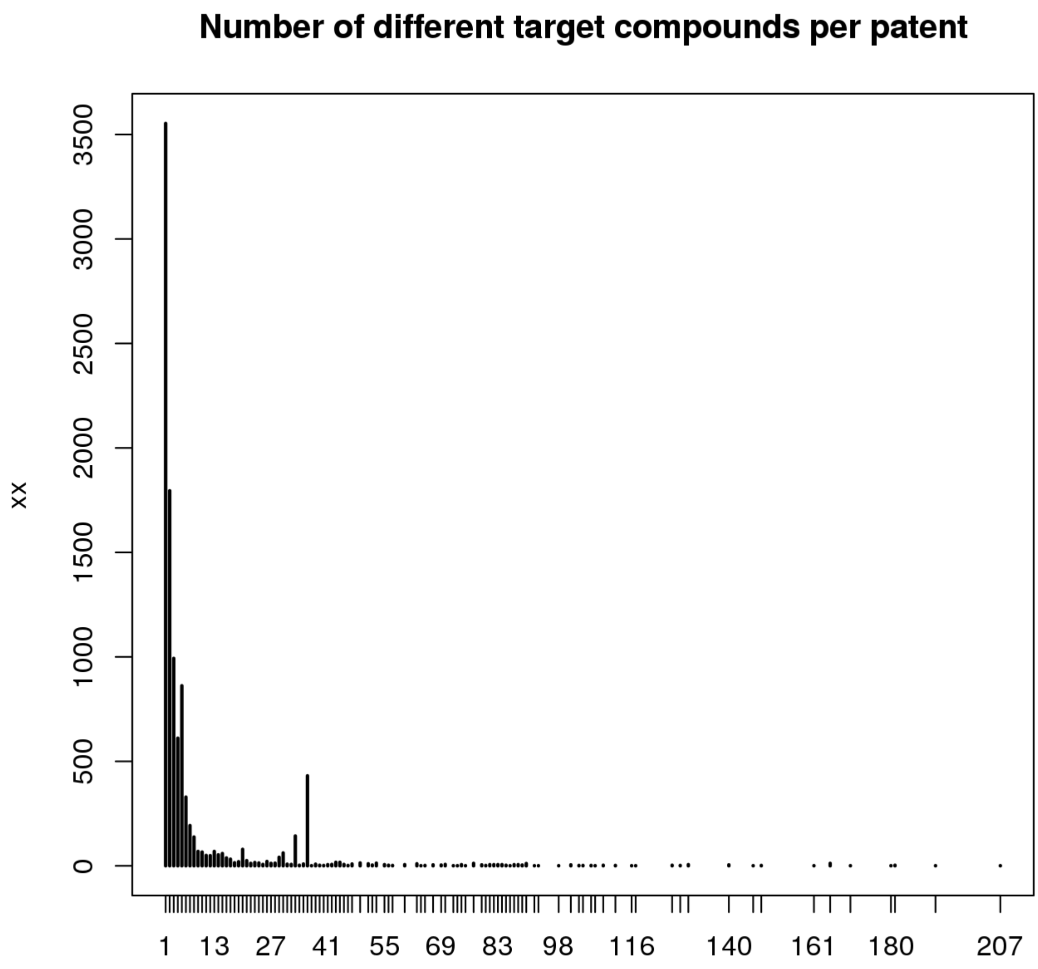


**Fig. S2.** Number of different target compounds per patent document.

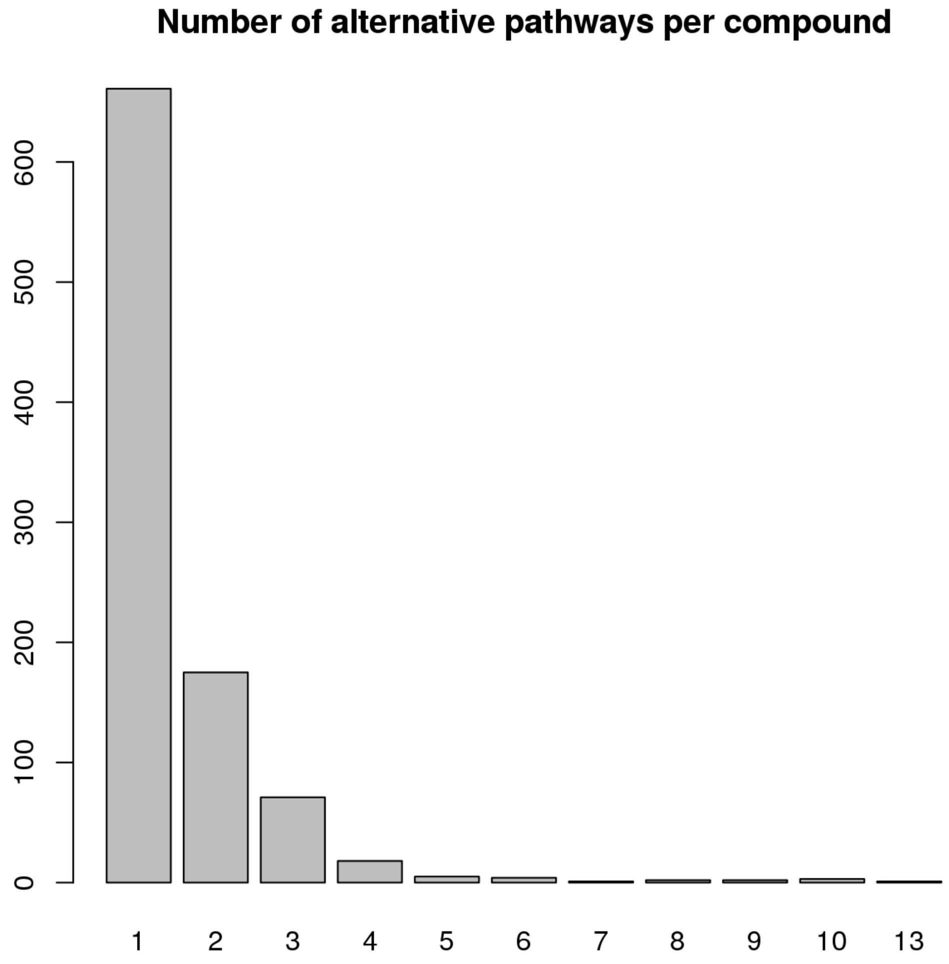


**Fig. S3.** Number of alternative pathways per compound.

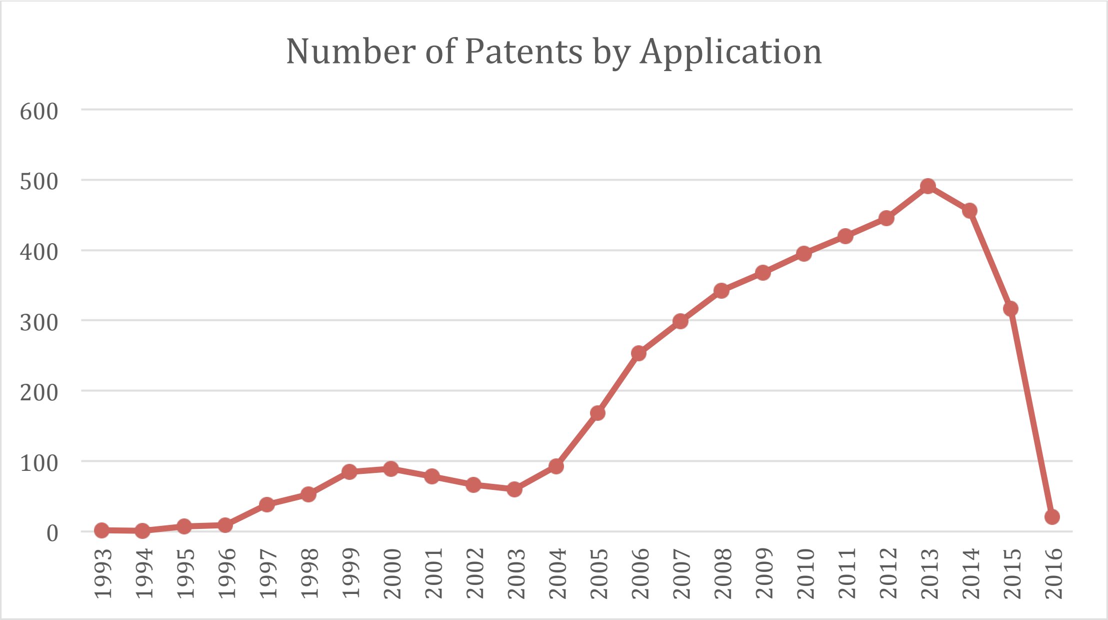


**Fig. S4.** Number of patents by application year.

Note: Source: own calculation based on the Derwent Innovation Index (DII). A decrease is noted in most recent period as there is a lag between patent application and inclusion in the DII.
